# Decentration and Tilt of Plate-Haptic Intraocular Lenses After Cataract Surgery: Anterior Segment Optical Coherence Tomography Evaluation

**DOI:** 10.7759/cureus.77634

**Published:** 2025-01-18

**Authors:** Takuya Kuriyama, Takashi Ono, Shuichiro Eguchi

**Affiliations:** 1 Ophthalmology, University of Tokyo, Tokyo, JPN; 2 Ophthalmology, Eguchi Eye Hospital, Hakodate, JPN

**Keywords:** cataract surgery, complications, intraocular lenses, visual acuity, visual function

## Abstract

Purpose: The aim of this study was to examine postoperative tilt and decentration after implantation of plate-haptic intraocular lenses (IOLs) after cataract surgery.

Methods: This was an observational retrospective study in which we analyzed the medical records of patients who underwent phacoemulsification with aspiration followed by the implantation of either a plate-haptic IOL (LS-313 MF15; Santen Pharmaceutical Co., Ltd., Osaka, Japan) or an open-loop IOL (SY60WF; Alcon Inc., Fort Worth, Texas, United States). We reviewed patient demographics, visual acuity, and manifest refraction prior to surgery, as well as at one day, one week, one month, and six months postoperatively. Anterior chamber depth, lens thickness, axial length, decentration, and tilt were measured using anterior segment optical coherence tomography.

Results: A total of 45 eyes from 24 patients were included in the study. The plate-haptic IOL group consisted of 23 eyes, with a mean age range of 75.0±7.8 years, while the open-loop IOL group comprised 22 eyes, with a mean age range of 75.3±6.9 years. Over the six-month period, the plate-haptic IOL group showed no significant changes in tilt or decentration. There were no differences in tilt or decentration between the groups at any observation point. In the plate-haptic IOL group, tilt one month after surgery showed a significant correlation with the preoperative tilt of the lens (p = 0.002).

Conclusion: The plate-haptic IOL group showed no significant increase in decentration or tilt at six months postoperatively. Plate-haptic IOL provided strong stability as an IOL platform.

## Introduction

The advancements in cataract surgery in recent years have emphasized both safety and the importance of excellent visual function. Accurate postoperative positioning of the inserted intraocular lens (IOL) is critical for maintaining high-quality visual function [1,2]. Previous studies have shown that IOL tilt or decentration can impair visual function by inducing optical aberrations and, in severe cases, reducing visual acuity [3]. Optical simulations suggest that a decentration of more than 0.5 mm can lead to notable visual symptoms [2]. Furthermore, minimizing IOL tilt reduces coma aberrations and enhances the quality of the retinal image [4,5].

Proper positioning of the IOL within the capsule is important not only for mono-focal IOLs but also for high-functioning IOLs, as even minor tilt or decentration can impair postoperative IOL function. Toric and multifocal IOLs are more sensitive to tilt and decentration than mono-focal IOLs [6], with decentration potentially leading to uneven light distributions between the distant and near foci [7]. Previous clinical studies have reported no significant differences in tilt and decentration between one-piece and three-piece IOL designs or between hydrophilic and hydrophobic IOLs [8,9]. A low-add-power, segmented, rotationally asymmetrical IOL is a novel plate-haptic IOL with a +1.5D asymmetry [10]. However, this add-on function can be compromised by IOL mispositioning, which can degrade vision quality. Although there have been reports on the effects of tilt and decentration on visual function in open-loop IOLs, few studies have evaluated postoperative tilt and decentration in plate-haptic IOLs [11], and no study has directly compared these parameters between open-loop and plate-haptic IOLs. Therefore, in this study, we compared the decentration and tilt of the plate-haptic and open-loop one-piece IOLs.

## Materials and methods

This was an observational clinical study conducted at the Eguchi Eye Hospital, Hakodate, Hokkaido, Japan. The study was approved by the Research Ethics Committee of the Eguchi Eye Hospital (approval number: 17000115) and adhered to the tenets of the Declaration of Helsinki. Informed consent was obtained from all participants through the opt-out method.

Inclusion and exclusion criteria

Patients who underwent phacoemulsification or implantation of either plate-haptic IOL (LS-313 MF15, LENTIS Comfort®; Santen Pharmaceutical Co., Ltd, Osaka, Japan) or open-loop IOL (SY60WF, Clareon®; Alcon Inc., Fort Worth, Texas, United States) for cataracts at the Eguchi Eye Hospital between October 2022 and January 2023 were included in the study. Patients with pseudoexfoliation syndrome, inflammatory eye disease, atopic dermatitis, a history of ocular trauma, or prior internal ocular surgery were excluded.

Data collection and intervention

Medical records were retrospectively reviewed for patients’ demographics, lens state, and examination data of the anterior chamber before surgery and one day, one week, one month, and six months after surgery. Data on anterior chamber depth, lens thickness, decentration, and tilt were collected using anterior segment optical coherence tomography (AS-OCT) (CASIA2; Tomey Corporation, Nagoya, Japan), and axial length was measured using swept-source OCT biometry (OA-2000; Tomey Corporation). Mydriasis was induced by topical instillation of 0.5% tropicamide and 0.5% phenylephrine hydrochloride (Mydrin-P; Santen Pharmaceutical Co., Ltd). After confirming sufficient mydriasis, AS-OCT images were captured by a skilled examiner with the eyelids fully open. Based on previous studies, tilt and decentration were measured using AS-OCT and its integrated software in eyes with IOL or cataracts [12].

Surgical procedure

The cataract surgery procedures were identical in both groups. All surgeries were performed under topical anesthesia using a 2.4 mm sclerocorneal incision after disinfection. The incision was made on the superior side in all cases. A continuous curvilinear capsulorhexis slightly smaller than the IOL optic diameter (approximately 5.5 mm) was performed in all cases. The SY60WF or LS-313 MF15 was implanted using a specialized injector. The haptics of the SY60WF were positioned at the 3 o’clock and 9 o’clock positions, while the haptics of the LS-313 MF15 were positioned at the 6 o’clock and 12 o’clock positions within the capsular bag. Levofloxacin (0.5%) was instilled four times daily for three days preoperatively. Postoperatively, 0.5% levofloxacin was instilled four times daily for one month. Betamethasone (0.1%) was administered four times daily for one week postoperatively, followed by 0.1% fluorometholone instilled four times daily for two months. Diclofenac (0.1%) was administered three times daily for three months postoperatively. All surgeries were performed by a single surgeon (SE). Regarding IOL specifications, the plate-haptic IOL (LS-313 MF15) is made of hydrophilic acrylic material. It has a total length of 11.0 mm and an optic diameter of 6.0 mm and contains a sector-shaped near visual acuity zone with +1.5D addition. The optic features a 360-degree squared sharp edge. On the contrary, the open-loop IOL (SY60WF) is made of hydrophobic acrylic material. The total length is 13.0 mm and the diameter of the optics is 6.0 mm. This IOL also has a square-edge lens design.

Statistical analysis

After testing for normality using the Kolmogorov-Smirnov test, the Mann-Whitney U test was used to compare age, axial length, anterior chamber depth, lens thickness, tilt, and decentration between the two groups. Categorical variables such as sex were compared using Fisher’s exact test or chi-square test. A mixed-effects model with Sidak’s multiple comparisons was used to compare tilt and decentration within each group. A linear regression model was applied to examine tilt and decentration before and one month after surgery. Statistical analyses were conducted using GraphPad Prism (Insightful Science, LLC, San Diego, California, United States). Statistical significance was defined at P < 0.05. All values are presented as mean ± standard deviation (SD).

## Results

A total of 45 eyes from 24 patients were included in the study. The plate-haptic IOL group consisted of 23 eyes (mean age, 75.0±7.8 years), and the open-loop IOL group consisted of 22 eyes (mean age, 75.3±6.9 years) (Table 1). Each patient received only one type of IOL; none had a plate-haptic IOL in one eye and an open-loop IOL in the other. Although there was no significant difference between the two groups in terms of age, sex, lens thickness, axial length, preoperative lens tilt, and preoperative decentration, the anterior chamber depth was shorter in the plate-haptic IOL group than in the open-loop IOL group (p = 0.004) (Table 1). No intraoperative or postoperative ocular complications were observed in either group.

**Table 1:** Demographic background of the patients who underwent cataract surgery Mann–Whitney U test was used to compare age, axial length, anterior chamber depth, lens thickness, tilt, and decentration between the two groups. Sex was compared using Fisher’s exact test. IOL: intraocular lens

	Plate-haptic IOL group	Open-loop IOL group	p-value
Number of eyes	23	22	-
Age (years), mean±SD (range)	75.0±7.8 (62-86)	75.3±6.9 (57-85)	0.873
Male:female (eyes)	4:19	9:13	0.108
Axial length (mm), mean±SD (range)	23.4±1.0 (21.7-26.2)	23.9±1.2 (21.8-25.4)	0.058
Anterior chamber depth (mm), mean±SD (range)	2.6±0.3 (2.1-3.0)	2.9±0.3 (2.3-3.3)	0.004
Lens thickness (mm), mean±SD (range)	4.6±0.5 (3.4-5.4)	4.5±0.4 (3.9-5.2)	0.149
Tilt (degree), mean±SD (range)	4.7±1.3 (2.2-6.5)	5.2±1.1 (2.5-7.1)	0.256
Decentration (mm), mean±SD (range)	0.13±0.04 (0.05-0.21)	0.16±0.09 (0.03-0.35)	0.412

**Figure 1:**
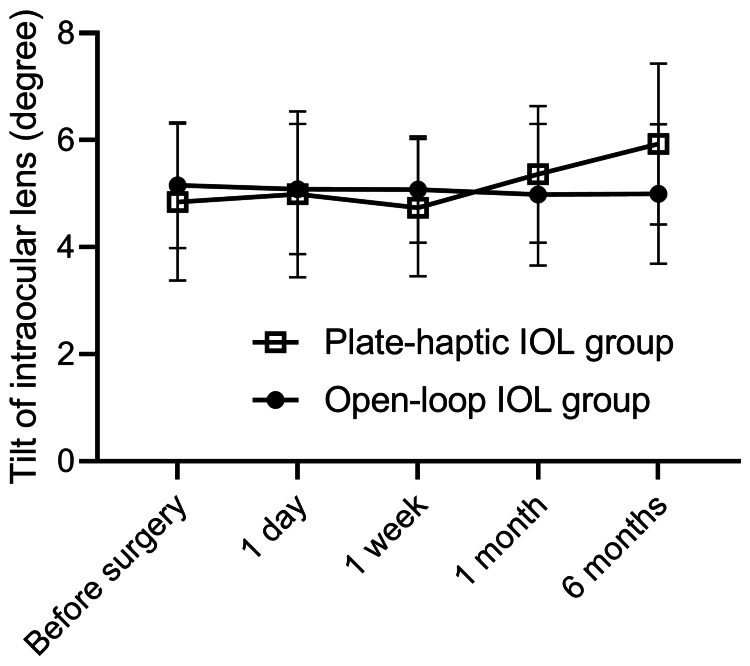
Tilt of plate-haptic and open-loop IOLs after cataract surgery There was no intragroup change in tilt before or after cataract surgery in either group. There was no difference in the tilt between the plate-haptic and open-loop IOLs. IOL: intraocular lenses

Regarding tilt, no intragroup changes were observed throughout the observation period in either group. Additionally, no significant differences were noted between the two groups at any time point (Figure 1).

**Figure 2:**
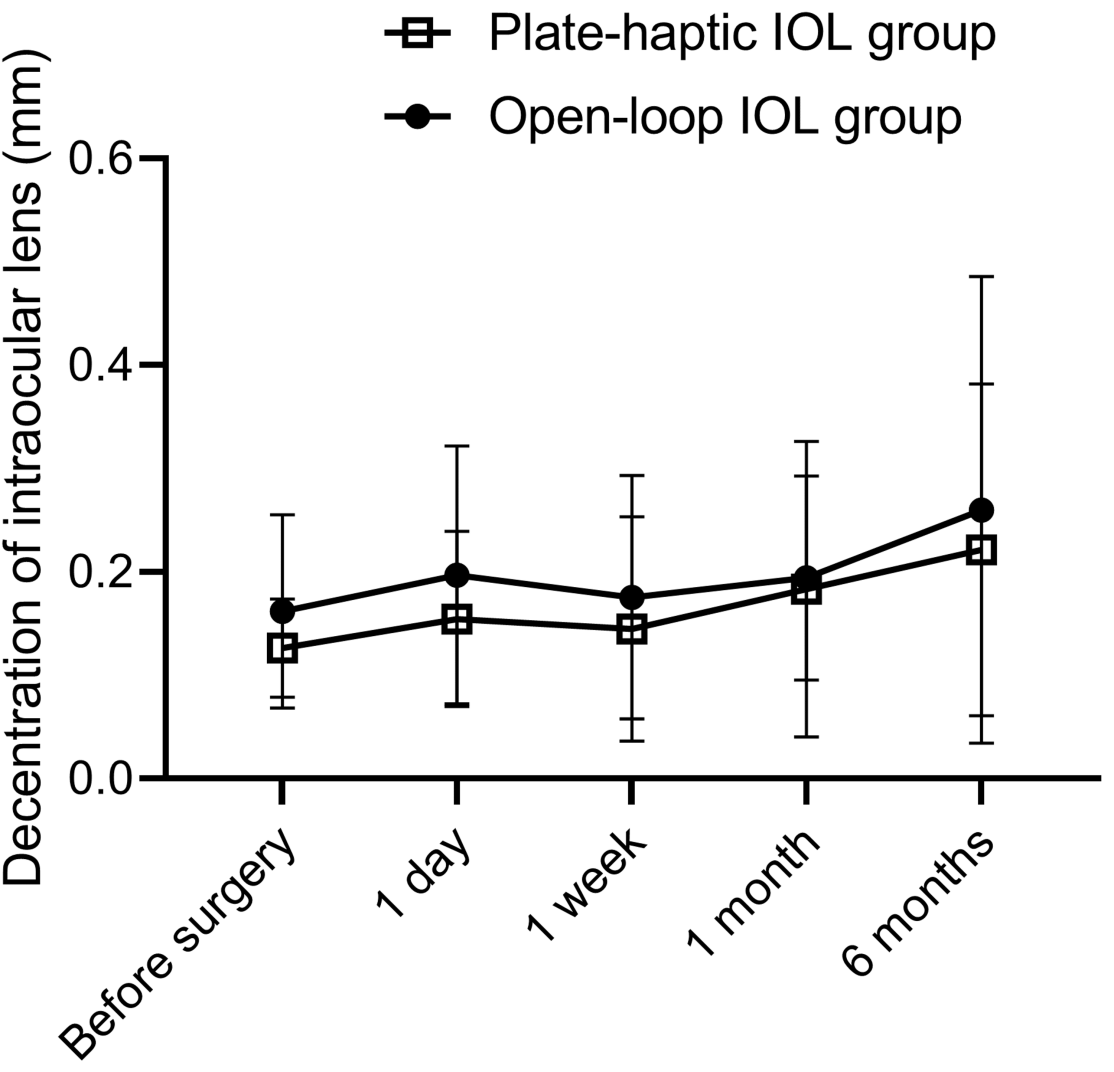
Decentration of plate-haptic and open-loop IOLs after cataract surgery There were no intergroup differences in the decentration of plate-haptic and open-loop IOLs. There was no intragroup change in decentration for six months in either group. IOL: intraocular lens

For decentration, no changes were observed in the open-loop IOL group and in the plate-haptic IOL group during the observational periods (Figure 2). No significant differences in decentration were found between the two groups at any time point.

**Figure 3:**
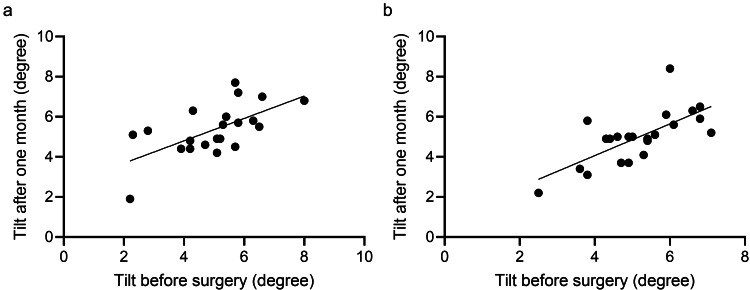
Relationship of tilt of lens before insertion of IOL and one month after cataract surgery (a) There was a significant positive relationship between the tilt of lens before and one month after insertion of plate-haptic IOL (p = 0.002, R^2^=0.393 Y=0.558X+2.569); (b) There was a significant positive relationship between the tilt of lens before and one month after insertion of open-loop IOL (p < 0.001, R^2^=0.489, Y=0.790X+0.908) IOL: intraocular lens

In the plate-haptic IOL group, tilt one month after surgery showed a significant correlation with the preoperative tilt values (p = 0.002, R^2^=0.393 Y=0.558X+2.569) (Figure 3a). A similar trend was observed in the open-loop IOL group (p < 0.001, R^2^=0.489, Y=0.790X+0.908) (Figure 3b).

**Figure 4:**
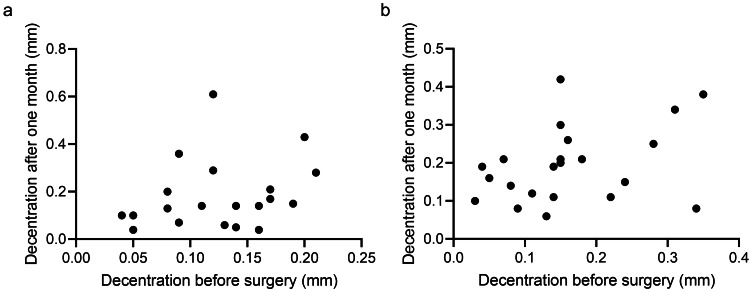
Relationship of decentration of lens before insertion of IOLs and one month after cataract surgery (a) There was no relationship between the decentration of lens before and one month after insertion of plate-haptic IOL; (b) There was no relationship between the decentration of lens before and one month after the insertion of open-loop IOL IOL: intraocular lens

In contrast, no significant correlation was found between decentration before and one month after surgery in either group (Figure 4).

Tilt orientation was predominantly oriented toward the inferior temporal side in both groups, while the decentration orientation was primarily oriented toward the temporal side in both groups (Table 2).

**Table 2:** Orientation of the tilt and decentration one month after cataract surgery The numbers shown indicate the percentage of intraocular lens tilt or decentration in each direction. Chi-square test was used to compare between groups. IOL: intraocular lens

		Superior temporal	Superior nasal	Inferior temporal	Inferior nasal	p-value
Tilt (%)	Plate-haptic IOL (%)	4.3	0.0	95.7	0.0	0.187
	Open-loop IOL (%)	18.2	0.0	81.8	0.0	-
Decentration (%)	Plate-haptic IOL (%)	30.4	8.7	34.8	17.4	0.671
	Open-loop IOL (%)	31.8	22.7	27.3	18.2	-

## Discussion

There was no difference in the tilt or decentration of the plate-haptic IOL compared with that of the open-loop IOL. It is important that the IOL be fixed consistently with the visual axis because misalignment of the IOL in the eye can result in increased higher-order aberrations, astigmatism, glare, halos, and reduced contrast sensitivity, thereby reducing visual function. Although decentration and tilt were inevitably observed six months after cataract surgery, these have been reported to be less clinically problematic at small values [13,14], and 2-3 degrees of tilt and about 0.2-0.3 mm of decentration have been reported to be clinically acceptable [15]. A recent report demonstrated that uneventful cataract surgery resulted in a mean tilt of 4 degrees toward the inferotemporal direction with 0.21 mm decentration of the IOL [8]. In our study, the postoperative tilt was 5.93 degrees, and the decentration was 0.22 mm in the plate-haptic IOL group after six months (Figures 1, 2); therefore, it was considered within the normal range. Our open-loop IOL group demonstrated the tilt was 4.99 degrees, the decentration was 0.26 mm, and the value was also in accordance with a previous report [8]. Thus, the shape of the IOL, whether plate-haptic or open-loop, did not affect the postoperative tilt and decentration. However, further long-term follow-up is needed because differences in the IOL location in the bag may change and be affected by the anterior-posterior deviation associated with bag shrinkage or the proliferation of lens epithelial cells in the bag.

IOL tilt and decentration are caused by various factors such as congenital misalignment of the capsular bag, larger preoperative tilt and decentration of the crystalline lens, shorter or longer axial length of the eye, surgical technique, and type of IOL. The type of IOL is particularly important. Aspherical IOLs can correct spherical aberrations; however, their function is limited when decentration or tilt increases [16]. Toric IOL also affects visual function when misalignment [17]. Furthermore, multifocal IOLs are more susceptible to decentration than mono-focal IOLs [18]. In our study, even though the tilt and decentration in the plate-haptic IOL cases were comparable to those in the mono-focal IOL cases and the values were within acceptable limits, indicating the usefulness of the plate-haptic IOL in the eye, the plate-haptic IOL group exhibited a short preoperative anterior chamber depth and a tendency toward short axial lengths, but we did not intentionally select eyes with these characteristics. This background may have influenced the outcome, as a longer axial length is associated with a greater IOL decentration [8].

The tilt and decentration of the crystalline lens tend to be larger towards the inferior temporal side, and the directions of tilt and decentration of the postoperative IOL are correlated with those of the preoperative crystalline lens [19]. Due to the misalignment between the axes of the cornea and lens, and the central fovea being positioned slightly temporal to the optical axis, the crystalline lens naturally exhibits tilt with decentration within the capsular bag. In our study, no differences were found in the postoperative orientation of tilt and decentration between the two groups. The tilt toward the inferior temporal side was the most frequently observed direction, which is consistent with previous reports [8,19]. Furthermore, the degree of the postoperative tilt was correlated with the preoperative tilt in the plate-haptic IOL group and the open-loop IOL group. Thus, patients with a significant crystalline lens tilt observed during the preoperative examination are likely to present with a large postoperative tilt. In these cases, it is pertinent to carefully calculate the IOL power, as the postoperative tilt could result in prediction errors.

Although previous reports have mainly used Purkinje or Sheimpflug imaging techniques [20], tilt and decentration were efficiently evaluated automatically using a corneal vertex with good repeatability [12,21]. AS-OCT is a noninvasive imaging technique that provides detailed tomographic images of the anterior eye segment at high resolution. AS-OCT provides a simple imaging method and a short imaging time, and changes in the anterior segment over time can be followed by multiple imaging modalities during follow-up after cataract surgery. To ensure reproducibility, multiple measurements were performed under identical conditions, and the best image without errors was adopted. It is also important to standardize the measurement conditions in the examination room because the position of the IOL may change depending on the pupil diameter and accommodation.

The current study has several limitations. First, it was difficult to evaluate the shape and position of the whole capsule of the crystalline lens before cataract surgery, and the measured values may have differed from the actual tilt and decentration. Second, the size of the continuous curvilinear capsulorhexis, which has been reported to be associated with IOL decentration [8], was not recorded. However, all surgeries were performed by a single skilled ophthalmologic surgeon without complications, and the capsulorhexis was considered uniform across cases. Third, owing to the retrospective nature of the study, the sample size was limited. Therefore, further prospective clinical studies are required.

## Conclusions

The plate-haptic IOL group showed no significant increase in decentration or tilt compared with the open-loop IOL group at six months postoperatively. In summary, the plate-haptic IOL provides strong stability as an IOL platform.
